# *N*^6^-methyladenosine modification—a key player in viral infection

**DOI:** 10.1186/s11658-023-00490-5

**Published:** 2023-10-12

**Authors:** Xiaoyue Zhang, Qiu Peng, Lujuan Wang

**Affiliations:** 1 Hunan Cancer Hospital and the Affiliated Cancer Hospital of Xiangya School of Medicine, Central South University, 283 Tongzipo Road, Changsha, 410013 Hunan China; 2 Department of Orthopaedics, The Second Xiangya Hospital, Central South University, Changsha, China; 3 Hunan Key Laboratory of Tumor Models and Individualized Medicine, The Second Xiangya Hospital, Central South University, Changsha, China; 4 Cancer Research Institute and School of Basic Medical Science, Central South University, Changsha, China

**Keywords:** m^6^A modification, DNA virus, RNA virus, Viral infection

## Abstract

*N*^6^-methyladenosine (m^6^A) modification is a dynamic, reversible process and is the most prevalent internal modification of RNA. This modification is regulated by three protein groups: methyltransferases (“writers”), demethylases (“erasers”), and m^6^A-binding proteins (“readers”). m^6^A modification and related enzymes could represent an optimal strategy to deepen the epigenetic mechanism. Numerous reports have suggested that aberrant modifications of m^6^A lead to aberrant expression of important viral genes. Here, we review the role of m^6^A modifications in viral replication and virus–host interactions. In particular, we focus on DNA and RNA viruses associated with human diseases, such as severe acute respiratory syndrome coronavirus 2 (SARS-CoV-2), human immunodeficiency virus (HIV)-1, Epstein–Barr virus (EBV), and Kaposi’s sarcoma-associated herpesvirus (KSHV). These findings will contribute to the understanding of the mechanisms of virus–host interactions and the design of future therapeutic targets for treatment of tumors associated with viral infections.

## Introduction

**Fig. 1:**
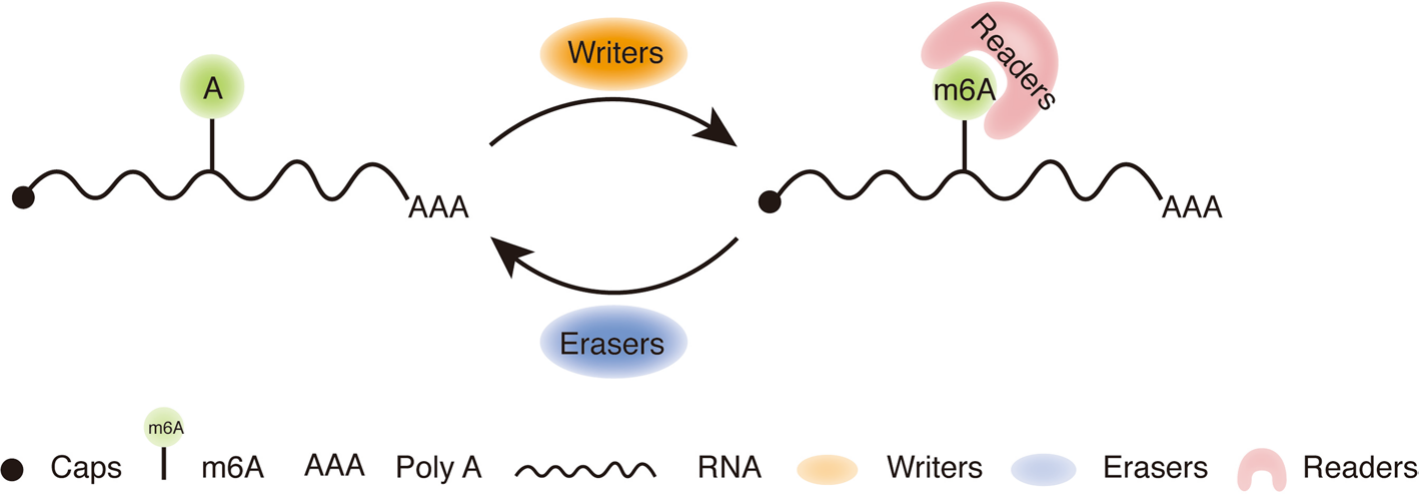
A model diagram of m^6^A modification. The m^6^A “writers” act as an important methyltransferase to write m^6^A modifications on mRNA. The “erasers” can erase m^6^A modifications from mRNA, prompting a dynamic and reversible modification process. The “readers” target m^6^A sites in a methylation-dependent manner, which reads m^6^A modifications to trigger a variety of biological processes

Since 1970, *N*^6^-methyladenosine (m^6^A) has been found to be the most popular internal modification of mRNA and noncoding RNA in eukaryotic cells, ranging from yeast, to *Arabidopsis thaliana* and *Drosophila*, to mammals; it is even found in RNA viruses [1, 2]. Following the development of m^6^A RNA immunoprecipitation and the basis of high-throughput sequencing, m^6^A RNA methylomes with ~ 100-nucleotide resolution were mapped. At the same time, more than 7000 mRNA and 300 noncoding (ncRNA) transcripts of human cells are involved in m^6^A. Meanwhile, m^6^A enrichment regions were revealed in 3′-UTR and around the stop codon [3]. The pathway identifying the factors from m^6^A regulation is made up of three distinct factors: the first type, named “writers,” that methylate adenosine at the N^6^ position, the second type, named “erasers,” that demethylate m^6^A for reversible regulation, and the last type, named “readers,” that act as direct sensors to monitor m^6^A site changes (Fig. 1).

A large heteromultimeric methyltransferase complex, also named m^6^A writer complex, wrestles to catalyze m^6^A deposition, in which methyltransferase-like 3 (METTL3)—a plainly catalyzed subunit—attaches methyl to adenosine residues accompanied by*S*-adenosyl methionine (SAM) activity [4–6]. Targeting *METTL3* by CRSIPR-Cas9 gene editing, experimental results prove that METTL3 is a major m^6^A methyltransferase in mESCs, as well as promoting ESC self-renewal [7]. Soon afterwards, studies found that methyltransferase-like 14 (METTL14) acts as a second methyltransferase, being an essential function to recognize the RNA substrate [8]. Meanwhile, on mammalian nuclear RNAs, METTL3 and METTL14 proteins can form a stable heterodimer core complex to deposit m^6^A in cellular [5]. In *Arabidopsis thaliana*, Wilm’s tumor 1-associating protein (WTAP) acts as a component for the methyltransferase complex to interact with METTL3 and METTL14. Furthermore, it is responsible for localizing nuclear speckles and catalyzing m^6^A methyltransferase activity [9]. Additionally, the “writer” complex has a “reserve army,” namely VIRMA, FLACC, RBM15, HAKAI, and RBM15/B, which are several assistant proteins that have a hand in correct positioning and functioning.

METTL3–METTL14 and WTAP, as an RNA m^6^A methyltransferase complex, mediate m^6^A modification of mRNA and noncoding RNA, as reviewed above. However, the process of m^6^A modification in human ribosomal RNA has not yet been studied. Recently, He et al. found that CCHC zinc finger-containing protein ZCCHC4, the first m^6^A methyltransferase found to mediate m^6^A methylation of eukaryotic, cytosolic rRNAs, methylates human 28S rRNA and is associated with a series of mRNA. Moreover, its function in regulating a variety of RNA-dependent biology is well known. In addition, methylation of m^6^A 4220 sites catalyzed by ZCCHC4 plays a central role in optimizing global translation activity, in which methylation affects cell proliferation and tumor growth [10]. m^6^A modification regulates the proliferation of a variety of tumor cells to promote tumorigenesis, such as bladder cancer [11], gastric cancer [12], colorectal cancer [13, 14], and lung cancer [15]. Mechanistically, m^6^A promotes tumor cell proliferation, migration, and invasion through regulating the m^6^A modification level of the target gene. Additionally, m^6^A can facilitate mRNA translation by interacting with the translation initiation machinery to promote tumor cell growth and survival. These studies demonstrate that m^6^A modification plays an important role in tumor cell proliferation and provide a research basis for tumor-targeted therapies.

Dynamic reversible m^6^A modification can be enzymatically removed by demethylase “eraser” enzymes. As far as we know, human obesity and energy homeostasis have been associated with fat mass and obesity-associated (FTO) protein [16]. With worldwide public health concerns and genome-wide associated studies, FTO has become mainstream in the research field [17]. In vitro studies performed in 2012 showed that in RNA the activity of FTO oxidative demethylation is related to *N*^6^-methyladenosine (m^6^A) residues, further validating that the major physiological substrate of FTO is m^6^A in nuclear mRNA [18]. The function of ALKBH5, a new mammalian RNA demethylase, is similar to that of FTO due to its demethylase activity targeting m^6^A in RNA. m^6^A in RNA. Recent research revealed that mRNA export regulation requires the participation of ALKBH5 protein in addition to RNA synthesis [19]. The C-terminus of ALKBH5 consists of Arg–Ser-rich regions, which may be disordered, that may control RNA interactions [20]. Surprisingly, ALKBH5 appears to localize to nuclear speckles, which may contribute to the assembly of mRNA processing factors. This phenomenon supports the idea that nuclear nascent RNAs are the main substrate for ALKBH5 [21].

**Fig. 2:**
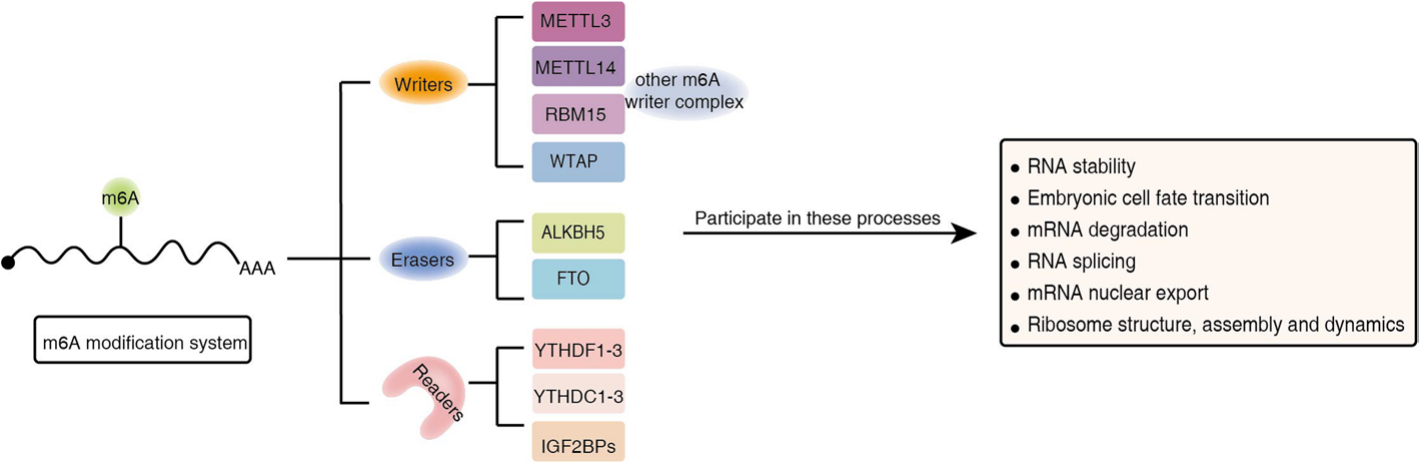
Schematic model illustrating the biological function with m^6^A modification. The modifications of m^6^A are regulated by “writers,” “erasers,” and “readers.” Some of the molecules related to m^6^A modification together with the biological functions in which they might be involved are listed in the model diagram

The human YTH family has five members, viz. YTH *N*^6^-methyladenosine RNA-binding proteins 1–3 (YTHDF1-3, also called DF1-3), YTH-containing structural domain 1 (YTHDC1/YT521-B, also called DC1), and YTH-containing structural domain 2 (YTHDC2, also called DC2). DF1-3 and DC2 are mainly localized in the cytoplasm, while DC1 is specifically localized in the nucleus [22–24]. Remarkably, He et al. measured by gel shift assay a higher affinity between YTH domain family and methylated probes, again showing that these proteins act as m^6^A-specific binders. [25] (Fig. 2).

During physiological or pathological processes, such as viral infectivity, the process by which m^6^A levels are regulated and the in vivo function of m^6^A in viral replication are largely unknown. It has been shown that m^6^A modifications and related enzymes play an important role in infecting host cells. The methylation pattern has also been elucidated in the transcripts of numerous DNA viruses such as KSHV [26], HBV [27], and EBV [28], and RNA viruses such as SARS-COV-2 [29], HIV-1 [30], and rotavirus [31]. Others have found that m^6^A modifications affect the expression of key genes involved in the viral life cycle. m^6^A modifications act as inhibitors or promoters of different types of pathogenic viruses. The next section reviews the biological function and the association of m^6^A modifications with DNA or RNA virus, and how this mechanism has an impact on viral biology.

## Biological function with m^6^A modification

The amount of studies on m^6^A methylation modification is innumerable, both here and abroad. Although research shows that m^6^A is involved in many biological processes, m^6^A locating in numerous transcripts has revealed that the specific biological functions are not systematically introduced, and its roles remain mysterious. Before RNA splicing or other RNA processing, m^6^A may exist in transcripts from previous work.With the combines MeRIP-seq and next-generation sequencing, m^6^A connected with non-coding RNA, introns, m^6^A-containing transcripts was found. Moreover, the evolutionary conservation and function of m^6^A have been revealed in embryonic stem cells [7]. Additionally, biological functions and associated regulatory proteins of m^6^A in cancer have also attracted much attention in recent years. These factors provide a possibility for us to study the biological functions related to m^6^A modification (Fig. 2).

### RNA stability and m^6^A modification

Studies by Wang and others have revealed that the RNA stabilizing protein HuR is connected with m^6^A demethylation, which is enough to maintain the ES cells in the ground state [32]. Of course, exploring m^6^A-dependent regulation of mRNA stability is essential in comparison with other gene expression regulation methods. IGF2BP, a new family of m^6^A readers, act to protect modified mRNAs from degradation, preferentially recognize m^6^A-modified mRNAs, and promote the stability of downstream target genes MYC in an m^6^A-dependent manner [33]. IGF2BP3 acts as a core m^6^A regulator in acute myeloid leukemia (AML) according to high-throughput library screening. Knockdown of IGF2BP3 significantly inhibits potential IGF2BP3 targets, specifically*RCC2* mRNA stability, with the attenuation of the malignant phenotype of AML [34].

### m^6^A regulates embryonic cell fate transition

The prevalence of *N*^6^-methyladenosine (m^6^A) modification in messenger RNAs was universally known. The fact that m^6^A regulates core pluripotency factors was revealed by Batista et al. m^6^A modification of target genes was erased by genetic inactivation or depletion of METTL3 in mice or humans. Further, the processing of ESC (embryonic stem cells) from self-renewal toward differentiation into several lineages was weakened in vitro and in vivo [7]. Here, cell cycle progression and differentiation of embryonic stem cells and tissue differentiation are inseparable from the strict and precise regulation of METTL3 [7–9, 35–37]. More interestingly, METTL3 function has infiltrated into neuronal differentiation and development of the nervous system, as well as into the process of learning and memory, including the circadian clock, cell progression, and maintenance of radial glial cells [36, 38–41].

### m^6^A modification with mRNA degradation

As far as we know, m^6^A modification is involved in mRNA processing. Research on the development of RNA methylation shows that m^6^A can bind to human YTH domain family 2 (YTHDF2), which is selective [42]. Thus, the regulation of mRNA degradation may be helped by m^6^A. YTHDF2 targets bound mRNA, binding in the mRNA decay sites. This pattern is similar to processing bodies. N-YTHDF2 localizes the YTHDF2-m^6^A–mRNA complex to a more specialized mRNA degradation machine of processing bodies to degrade SON mRNA [42]. On the other hand, it is suggested that mRNA attenuation caused by m^6^A modification is not due to the binding of YTH protein but occurs through the miRNA pathway [32].

### m^6^A modification affecting RNA splicing

There is no denying that FTO (a demethylase enzyme “eraser”) can govern gene expression and mRNA splicing of grouped genes according to m^6^A-seq and transcriptome analyses. Mainly, when regulating m^6^A levels neighboring splice sites, the exonic splicing of regulatory factor RUNX1T1 is dominated by FTO [43]. Interestingly, m^6^A takes part in splicing when RSV-infected chicken embryo fibroblasts treated with cycloleucine can inhibit internal methylation. Under these circumstances, it also can increase the level of unspliced viral RNA and reduce the level of spliced RNA . The above exposition was proved by Stoltzfus and Dane’s research [44]. The further significance of m^6^A modification in distinct RNA splicing has continued to be explored.

### mRNA nuclear export under m^6^A modification

After mRNA undergoes splicing, it faces the end of nucleus export, and is translated or degenerated in the cytoplasm. Studies by Camper and others found that there was no change to nonpolyadenylated RNA in the nucleus, although the nuclear mRNA residence time increased by 40% under the control of STH-treated HeLa cells. These results make clear that m^6^A modification plays a regulatory role in the whole mechanism, but it is not used in mRNA nuclear transport itself [45]. 5-Bromouridine (BrU) incorporation followed by immunofluorescence analysis showed that nascent RNA is distributed in the cytoplasm. It is indisputable that the level of RNA in the cytoplasm increased significantly, which accelerates nuclear RNA export in ALKBH5-deficient cells. ALKBH5 can affect mRNA level; in other words, mRNA is an important part of ALKBH5 [21].

### m^6^A affects ribosome structure, assembly, and dynamics

The chemical modification of human rRNA contains about 210 sites, most of which are 2′-O methylations (Nm) and pseudouridines (Ψ) [46]. Two m^6^A modifications of 28SrRNA at position 4220 and 18S rRNA at position 1832 are known to exist in rRNA [46–48]. The structure, assembly, and dynamics of ribosomes are usually influenced by various modifications of rRNA [49, 50]. Whether the regulatory effect of rRNA modification on translation level can potentially affect human health is the focus of attention. He et al. found that new rRNA-modified enzymes are groundbreaking in explaining additional translation regulation mechanisms. ZCCHC4, a new m^6^A methyltransferase, regulates ribosomal subunit levels, which is an important intracellular biological function. When the *ZCCHC4* gene was knocked out by CRISPR-Cas9, the level of mature rRNA was not significantly changed, but the peak of ribosomal subunit in 60S was significantly lower than the 80S peak in *ZCCHC4*-knockout cells compared with wild-type cells, as shown by structural analysis and using the established method [10].

## m^6^A modifications engage in viral infection: the role of writers, erasers, and readers

**Fig. 3:**
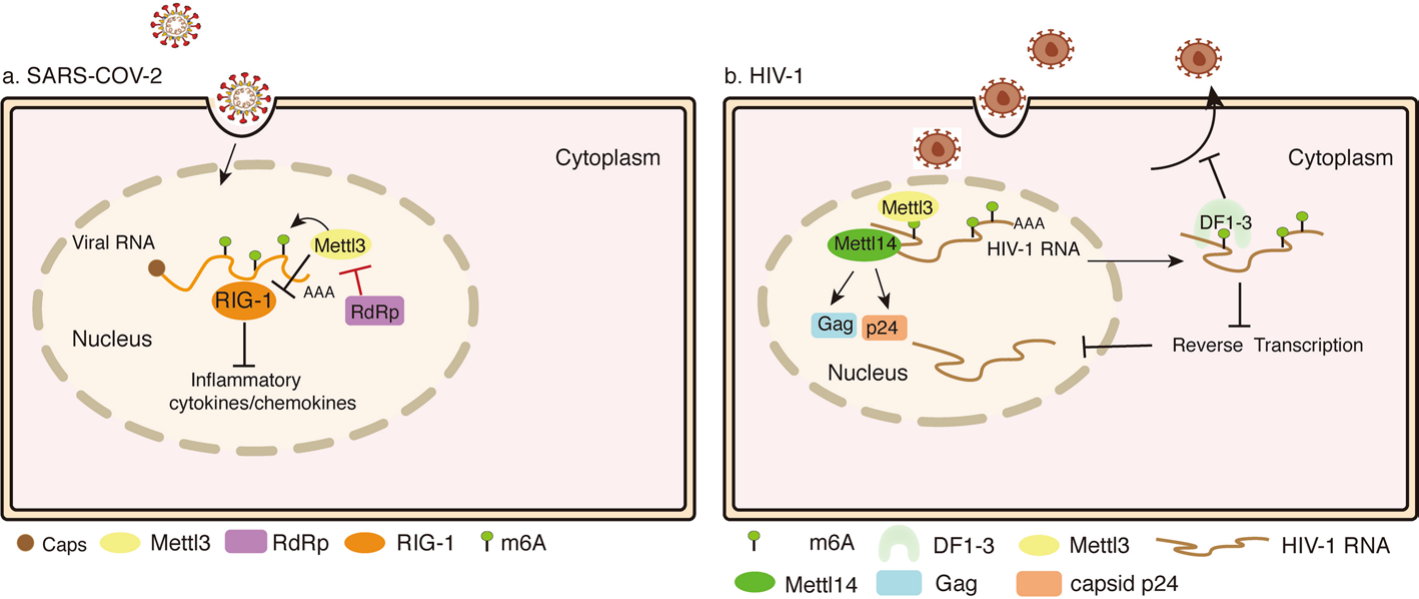
Schematic representation of m^6^A modification in RNA virus infection. **A** METTL3 promotes m^6^A modification of SARS-CoV-2 RNA and proviral gene expression. METTL3 inhibits innate immune signaling effector molecule RIG-I binding to SARS-COV-2 RNA. METTL3 interacts with the N region of RdRp (a viral RNA polymerase), and RdRp inhibits the sumoylation and ubiquitination modifications of METTL3 through posttranscriptional modifications. **B** The m^6^A writers METTL3 or METTL14 promote Gag protein expression and the level of capsid p24 release. YTHDF1-3 proteins bind with HIV-1 RNA to negatively regulate HIV-1 post-entry infection by blocking viral reverse transcription in CD4^+^ T cells

**Fig. 4:**
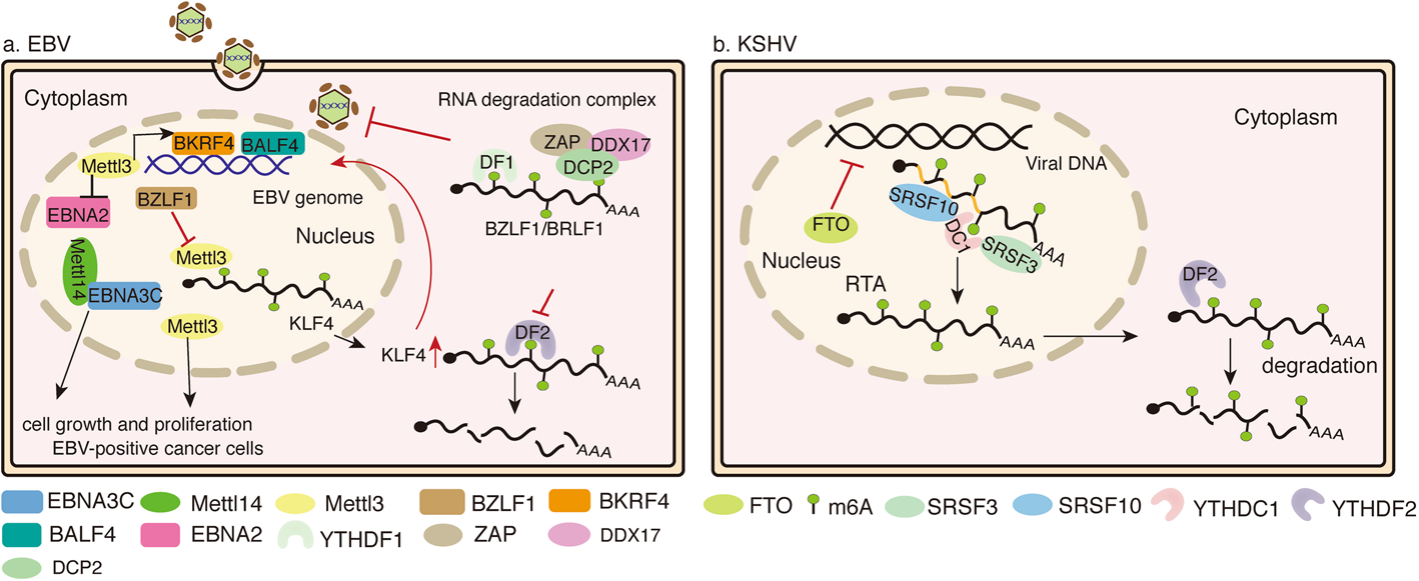
Schematic representation of m^6^A modification in DNA virus infection. **A** The EBV latent antigen EBNA3C upregulates the level of the methyltransferase METTL14. EBNA3C cooperates with METTL14 to promote cell growth and proliferation. BZLF1 repressed METTL3 expression by binding to the promoter. However, low expression of METTL3 repress the host gene KLF4. Conversely, high expression of KLF4 promotes BZLF1 expression and lytic infection of EBV in epithelial cells. METTL3 promotes the production of progeny virions and expression of the late viral lytic protein BKRF4, BALF4 in Akata cells. Furthermore, METTL3 enhances EBNA2 expression. YTHDF1 promotes the binding of RNA degradation complexes (ZAP, DDX17, and DCP2) to the mRNAs of BZLF1 and BRLF1 to suppress EBV infection and replication. **B** The m^6^A demethylase FTO decreases the levels of m^6^A and enhances TPA induction of KSHV lytic gene expression. m^6^A nuclear reader protein YTHDC1 and the related splicing factors SRSF3 and SRSF10 bind to several m^6^A sites on the RTA (a key KSHV lytic switch protein) pre-mRNA, which are essential for its splicing. In the cytoplasm, YTHDF2 mediates degradation of KSHV transcripts, leading to repression of viral replication

Recently, more and more strong evidence supports such a hypothesis that viruses, including KSHV, EBV, etc. and even lytic RNA viruses in the cytoplasm, have formed complex, highly evolutionary, and coordinated regulatory networks to achieve “self-serving” purposes. This is mainly to utilize and regulate the host’s epigenetic genome to intervene in the process of innate immunity against viruses in the host, thereby promoting virus replication, virus production, and pathogenesis. In addition, researchers have found that it is possible that antiviral innate immune responses could be regulated through RNA modification, the first response provided by the innate immune system to resist virus infections. However, if the presence of nucleotide modifications in RNA is known to correlate with crippled innate immune signaling, then the complex and multifaceted story behind the scenes has not been explored. In summary, there is no detailed description of these aspects at present, so it is summarized in this review (Figs. 3, 4).

### m^6^A modification in SARS-CoV-2

SARS-CoV-2 is an RNA virus that causes severe acute respiratory syndrome and is the causative agent of coronavirus disease 2019 (COVID-19). The first study used the nanopore-based direct RNA sequencing (DRS) approach to study the SARS- CoV-2 transcriptome from Kim et al. [51]. m^6^A is widely distributed and dynamically regulated in the positive-sense genome and in negative-sense RNA intermediates [29]. The m^6^A modification is enriched in the nucleocapsid (N) region of the viral genome according to liquid chromatography-tandem mass spectrometry. Deficiency of METTL3 reduces m^6^A modification, viral load, and proviral gene expression of SARS-CoV-2 RNA [52]. The catalytic activity of METTL3 is required for the synthesis of viral RNA within 24 h postinfection [53]. However, an interesting study revealed that METTL3 interacts with the N region of RdRp (a viral RNA polymerase), and RdRp inhibits the sumoylation and ubiquitination modifications of METTL3 through posttranscriptional modifications [54]. RBM15, a “writer” that recruits methyltransferase complexes to target genes, promotes methylation of adenosine nucleotides [6]. RBM15 regulates the expression of multiple target genes (such as CASP1, CASP5, and TRIB1) by upregulating the level of m^6^A modification, exacerbates the inflammatory response, and promotes the cell death signaling pathway in COVID-19 [55]. According to these studies, m^6^A modifications play a dual role in promoting or inhibiting SARS-CoV-2 replication, providing a foundation to investigate host–virus interactions.

According to the newest epidemiologic data, various mutations in the SARS-CoV-2 viral genome have resulted in the formation of new viral strains. Among them, seven SARS-CoV-2 subtypes have attracted the most attention. The World Health Organization (WHO) (https://www.who.int/en/activities/tracking-SARS-CoV-2-variants) named them Alpha (B.1.1.7), Beta (B.1.351), Gamma (P.1), Delta (B.1.617.2), and Omicron (B.1.617.2) subtypes, which are now designated as variants of concern (VOC) (B.1.1.529) [56]. Three different SARS-CoV-2 variants, B.1, B.1.1.7, and B.1.351, infected Vero cells with loss of the m^6^A peak in RNA that was more noticeable in B.1 and B.1.1.7 infections compared with the B.1.351 infection. During SARS-CoV-2 infection, METTL3 partially relocated from the nucleus to the cytoplasm. Importantly, the cytoplasmic localization of METTL3 was more pronounced in the B.1 and B.1.1.7 variants compared with the B.1.351 variant and had an impact on the formation of the METTL3/METTL14 functional complex in infected cells, which may also explain the overall reduction in the m^6^A modification in the infected cells [57, 58]. RNA methylation at the adenosine nitrogen-6 position (m^6^A) is known to be altered in cells infected with SARS-CoV-2. Recent studies have shown that direct RNA sequencing comparison of variants Beta and Eta, from Brazilian SARS-CoV-2 samples, reveals a fourth C > U change at 28,884b in DRACH that may affect methylation [59]. In addition, Batista-Roche et al. sequenced nasopharyngeal samples from SARS-CoV-2-positive outpatient and hospitalized individuals aged 30–60 years and found significant differences in global m^6^A methylation in VOC Alpha, Gamma, Delta, and Omega, variant of interest Epsilon, and B.1.1.519 lines [60].

Moreover, m^6^A modification is an important mechanism for exogenous RNA evasion of host immune response events [61–64]. Surprisingly, m^6^A localization and methylation motifs of host cells were altered after SARS-CoV-2 infection [29]. Cellular transcripts involved in establishing an antiviral immune response are posttranscriptionally regulated by m^6^A modification [65–67]. Knockdown of METTL3 increases the expression of innate immune signaling effector molecules in host cells and promotes RIG-I binding to SARS-CoV-2 RNA. Mutation of m^6^A modification sites on SARS-CoV-2 RNA increases RIG-I recognition and expression of inflammation-related genes [52]. There is a probability that SARS-CoV-2 infection leads to changes in the m^6^A modification status of host cell transcripts, which may be induced directly by the virus or through the cellular response to infection (Fig. 3a).

### m^6^A modification in HIV-1

With the in-depth study of virology, the life cycle of most viruses has some common characteristics that can be roughly divided into three important stages: (1) recognition, attachment, and entry of viral receptors; (2) expression and synthesis of viral genes and genome replication; (3) virion assembly and release. Throughout these prolonged periods, the genomic RNA and mRNAs of multiple viruses have been marked with m^6^A dynamic modification, although the precise functional importance of m^6^A in the life cycle remains unclear [68–73].

Three studies found that m^6^A modification had some regulatory mechanisms in the viral genome and replication process in 2016, all using MeRIP-seq or PA-m^6^A-seq to identify specific m^6^A methylation sites on HIV-1 RNA [30, 74, 75]. *N*^6^-methyladenosine modification and related proteins participating in the m^6^A pathway play an indispensable role in limiting or regulating the life cycle of certain viruses. On the basis of immunoprecipitation with poly(A)-enriched RNA, multiple m^6^A modification sites exist in the HIV-1 RNA genome according to the use of m^6^A-special antibodies. In HIV-1-positive cell lines, the m^6^A writers METTL3 or METTL14 promote Gag protein expression and the level of capsid p24 release. Studies have indicated that YTHDF1-3 proteins bind with HIV-1 RNA to negatively regulate HIV-1 post-entry infection by blocking viral reverse transcription. In addition, endogenous YTHDF1-3 proteins play the same role to inhibit HIV-1 post-entry infection in CD4^+^ T cells [75]. Beyond the above studies, two aimed studies to understand the effect of m^6^A on HIV-1 infection.

The modification of m^6^A increases a new regulatory layer of posttranscriptional gene expression that is related to the response of T cells to HIV infection, the production of interferon I, and the differentiation and homeostasis of T cells. The methylation of host and viral were plentiful in HIV-infected T cells. In-depth studies have shown the mechanism by which m^6^A methylation promotes viral replication. Additionally, studies have revealed that methylation at the A7883 site of HIV-1 Rev response element (RRE), an RNA element within the HIV-1 env gene and a switch regulating nuclear export of virus RNA, enhanced the RRE complex’s formation and HIV gene expression [30] (Fig. 3b).

### m^6^A modification in influenza A virus

The internal adenosine residue of influenza virus mRNA is transcriptionally methylated to form *N*^6^-methyladenosine (m^6^A). Influenza A virus (IAV) is an enveloped virus whose genome consists of eight single-stranded negative-sense RNA fragments encoding up to 18 proteins [76]. The first virus found to express mRNA-containing internal m^6^A residues was influenza A virus (IAV). Various mRNAs of this virus were reported to contain approximately 24 m^6^A residues. Among them, the HA mRNA fragment is the one with the highest number of m^6^A residues, eight of which were detected by biochemical analysis [73, 77].

In the human lung epithelial cell line A549, METTL3 mutant inactivation inhibits IAV replication, whereas ectopic overexpression of YTHDF2 increases IAV replication and infectious particle production. This may be due to the fact that YTHDF2-mediated mRNA degradation reduces host antiviral gene transcripts and thus promotes viral replication. IAV viral mRNAs encoding the structural proteins HA, NA, M1/M2, and NP have high levels of m^6^A addition, but low levels of m^6^A are found in mRNAs encoding the viral polymerase proteins PB2, PB1, and PA [78]. m^6^A is involved in the regulation of the IAV mRNA splicing process. YTHDF1 binds to the influenza A virus NS1 protein 3′ splicing site to inhibit NS splicing and promote IAV replication and pathogenicity in vitro and in vivo [79].

### m^6^A modification in EBV replication

Epstein–Barr virus (EBV), a gamma herpesvirus, is a ubiquitous cause of infection in humans worldwide [80]. The m^6^A modification is particularly important for EBV-mediated cell transformation and tumorigenic activity from the virus, which will make an invaluable contribution to clinical targeted drug research. Lang et al. demonstrated m^6^A modification in viral transcripts during the latent and lytic phases of EBV infection. The EBV latent antigen EBNA3C upregulates the level of the methyltransferase METTL14. EBNA3C cooperates with METTL14 to promote cell growth and proliferation. Knockdown of METTL14 decreased the tumorigenic activity of EBV-transformed cells in a xenograft animal model system [81].

After acute EBV infection, BZLF1, a highly expressed gene, repressed METTL3 expression by binding to a promoter. m^6^A modification of the host gene KLF4 is repressed by low METTL3 expression. However, knockdown of YTHDF2 increased KLF4 mRNA stability. Conversely, high expression of KLF4 promotes BZLF1 expression and lytic infection of EBV in epithelial cells. This will provide a feedback loop to promote EBV infection in host cells [82]. Knockdown of METTL3,DAA, or UZH1a reduced the production of progeny virions and expression of the late viral lytic protein BKRF4, BALF4 in Akata cells. In the EBV lytic cycle, knockdown of METTL3 was essential to inhibit the growth and survival of EBV-positive cancer cells, but did not affect EBV-positive cancer cells that did not undergo lytic activation [28]. Zheng et al. found that EBNA2 and BHRF1 contain m^6^A modifications in EBV infections. Knockdown of METTL3 suppressed EBNA2 expression [83]. Xia et al. revealed the presence of EBV transcripts modified by m^6^A in nasopharyngeal carcinoma cells, B cells, and tissue samples. YTHDF1 suppressed the expression of viral immediate-early (IE) lytic genes BZLF1 and BRLF1 by reducing the mRNA half-life. In addition, YTHDF1 promotes the binding of RNA degradation complexes (ZAP, DDX17, and DCP2) to the mRNAs of BZLF1 and BRLF1 to suppress EBV infection and replication [84] (Fig. 4a).

### m^6^A modification in KSHV replication

Recent studies have examined the biological significance of RNA m^6^A modification for Kaposi’s sarcoma-associated herpesvirus (KSHV). m^6^A nuclear reader protein YTHDC1 and the related splicing factors SRSF3 and SRSF10 bind to several m^6^A sites on the RTA (a key KSHV lytic switch protein) pre-mRNA that are essential for its splicing. Inhibition of the m^6^A demethylase FTO increased the levels of m^6^A and enhanced TPA induction of KSHV lytic gene expression. Knockdown of METTL3 has the opposite effect [85], proving that the lytic gene expression and replication are manipulated by KSHV utilizing host m^6^A-modified machinery. Knockdown of YTHDF1, YTHDC1, or YTHDC2 had no significant or consistent effect on viral lytic replication [26]. Knockdown of YTHDF2 led to a fourfold increase in KSHV production and an increase in RTA, ORF57, ORF-K8, and ORF65 lytic transcripts through an increase of the half-life of KSHV transcripts [86]. Since KSHV has a complex temporal lytic replication cycle, YTHDF2 and other reader proteins may prefer distinct sets of viral transcripts at different time points. What is even more compelling is that Hesser et al. confirmed that m^6^A machinery plays two roles: “good guys” and “bad guys,” by promoting the expression of Kaposi’s sarcoma-associated herpesvirus (KSHV) virus genes and supporting resistance to KSHV virus gene expression dependent on different cell types, respectively [86] (Fig. 4b).

## m^6^A modifications as therapeutic targets in the viral life cycle

Despite numerous pieces of evidence suggesting the involvement of m^6^A modifications in regulating the viral life cycle and tumor progression, as well as the rationale for targeting epigenetic regulators in cancer therapy, only a few epigenetically targeted drugs have reached the clinical trial stage [87]. Overall, the development of m^6^A regulator-targeted small molecule drugs is still in its infancy. Existing inhibitors focusing on FTO, METTL3, are a promising strategy for cancer therapy. However, specific inhibitors of m^6^A readers, including YTHDFs, have not been reported.

Elvitegravir, an FDA-approved drug originally developed for the treatment of human immunodeficiency virus (HIV) infection, increased STUB1-mediated proteasomal degradation of METTL3 and significantly inhibited esophageal squamous cell carcinoma (ESCC) invasion and metastasis [88]. HBV is a member of the *Herpesviridae* family. The genome of HBV is a circular, partially double-stranded DNA genome that replicates by reverse transcription of pregenomic RNA. Hepatitis B virus pregenomic RNA (HBV pgRNA) promotes proliferation, stemness, and tumorigenicity of HCC cells through upregulation of the expression of the m^6^A reader IGF2BP3 at the posttranscriptional level. Interferon (IFN)-α-2a decreases the stability of pgRNA by increasing its *N*^6^-methyladenosine (m^6^A) RNA modification. These results suggest that HBV-associated hepatocellular carcinoma (HCC) may be suppressed by modulating m^6^A modifications [89].

Human β-coronaviruses HCoV-OC43 and SARS-CoV-2 show similar dependence on host m^6^A methyltransferase and cytoplasmic m^6^A reader proteins in their productive growth. Infection with HCoV-OC43 alters YTHDF1 and YTHDF3 subcellular localization from the cytoplasm to aggregate in the nucleoplasm. Infection with HCoV-OC43 alters YTHDF1 and YTHDF3 subcellular localization from the cytoplasm to aggregate in the nucleoplasm. Coronavirus RNA synthesis occurs in double-membrane vesicle structures called replication organelles (Ros) in the cytoplasm [90]. The METTL3 inhibitor STM2457 inhibits double-stranded RNA (dsRNA) aggregation in Ros and N protein accumulation in the cytoplasm. Additionally, STM2457 produced antiviral effects against HCoV-OC43 or SARS-CoV-2 that were independent of the enhancement of type I IFN signaling. Thus, there is an important role for the β-coronavirus replication cycle in suppressing the catalytic activity of METLL3 [53]. The second-generation structurally unique METTL3 inhibitor STM3006 leads to dsRNA formation and a robust cell-intrinsic interferon response that stimulates antitumor immunity. One of the major consequences of METTL3 inhibition is the stabilization of newly synthesized transcripts. This is a complementary mechanism of action, distinct from current immunotherapeutic agents that target immune checkpoints such as the PD1/PDL-1 axis, CTLA4, or LAG3. STC-15 is another METTL3 inhibitor with comparable potency to STM3006, with improved oral bioavailability and metabolic stability, and is currently being evaluated in a phase I clinical trial in solid tumors (NCT05584111) [91].

In recent years, oncocytic viruses (OVs) have been used to guide tumor-targeted therapy or immunotherapy. Newcastle disease virus (NDV) is a solid tumor lysing agent that directly kills tumor cells and increases exposure to tumor antigens, triggering the proliferation of tumor-specific immune cells, thereby enhancing the efficacy of NDV against cancer [92]. NDV infection triggers an increase in RNA m^6^A modifications in vivo and in vitro. m^6^A modification localization in mRNAs is altered following NDV infection. The number of m^6^A peaks increases in the coding region and decreases in the stop codon and 3′-UTR regions. m^6^A writers and erasers dynamically regulate the NDV life cycle. Overexpression of ALKBH5 and FTO significantly increases viral titer, NDV RNA levels in cellular supernatants, and NDV N protein expression, whereas METTL3, METTL14, and WTAP overexpression decreased viral titer. Importantly, host genes undergo varying degrees of dynamic m^6^A modification following NDV infection [93]. Targeting m^6^A modification molecules could be an effective measure to enhance the therapeutic efficacy of NDV on cancer.

## Conclusions and prospects

Viruses are dependent on host cells for their entire life process, either directly using host cells for their own vital events, or adjusting and destroying host cells so that the host does not work properly, indirectly promoting its own life cycle. Summing up the above research, we can easily see that the members of the m^6^A system, i.e., “writers,” “readers,” and “erasers,” have established clear accountability and shared responsibility in virus infection. According to the above-mentioned reports, the role, function, and mechanism of key molecules in the m^6^A system in different viral infections are summarized in Table 1. In addition, these conclusions can be used for reference to further study the effect of viral infection on m^6^A modification and the function in virus–host interactions. In fact, the long-term struggle between virus and host during the process of evolution has led to a highly complex immune system and antivirus infection mechanisms in organisms.

**Table 1:** Mechanism of m^6^A modification occurring in viruses

Type	Virus	Protein	Biological function	Refs.
	SARS-CoV-2	METTL3	Enhance m^6^A modification of SARS-CoV-2 RNA, viral load, and proviral gene expression	[52]
			Interacts with the N region of RdRp	[54]
			Inhibits RIG-I binding to SARS-CoV-2 RNA	[52]
		RBM15	Regulates the expression of multiple target genes	[55]
RNA virus			Exacerbates the inflammatory response and promotes cell death signaling pathway in COVID-19	
	HIV-1	METTL3	Promotes Gag protein expression and the level of capsid p24 release	[75]
		METTL14		
		YTHDF1-3	Binds with HIV-1 RNA to negatively regulate HIV-1 post-entry infection by blocking viral reverse transcription	
	KSHV	YTHDC1	Binds to m^6^A sites on the RTA pre-mRNA	[85]
		FTO	Decrease m^6^A level and enhanced TPA induction of KSHV lytic gene expression	
		YTHDF2	Decrease the half-life of KSHV transcripts	[86]
	EBV	METTL14	Cooperates with EBNA3C to promote cell growth and proliferation	[81]
DNA virus			Increases the tumorigenic activity of EBV-transformed cells	
		METTL3	BZLF1 represses METTL3 expression by binding to promoter	[82]
			Promotes the production of progeny virions and expression of the late viral lytic protein BKRF4, BALF4	[28]
			Promotes EBNA2 expression	[83]
		YTHDF1	Promotes the binding of RNA degradation complexes (ZAP, DDX17, and DCP2) to the mRNAs of BZLF1 and BRLF1 to suppress EBV infection and replication	[84]
		YTHDF2	Increases KLF4 mRNA instability	[82]

*SARS-CoV-2* severe acute respiratory syndrome coronavirus clade 2, *RBM15* RNA-binding motif protein 15, *RdRp* RNA-dependent RNA polymerase

## Data Availability

Not applicable.

## Abbreviations

m^6^A: *N*^6^-methyladenosine
SAM: *S*-adenosyl methionine
ncRNA: Non-coding RNA
METTL3: Methyltransferase-like 3
WTAP: Wilm’s tumor 1-associating protein
FTO: Fat mass and obesity-associated
YTHDC1: YTH-containing structural domain 1
AML: Acute myeloid leukemia
ESC: Embryonic stem cells
RRE: Rev response element
EBV: Epstein–Barr virus
KSHV: Kaposi’s sarcoma-associated herpesvirus
IAV: Influenza A virus
ESCC: Esophageal squamous cell carcinoma
HBV pgRNA: Hepatitis B virus pregenomic RNA
NDV: Newcastle disease virus
dsRNA: Double-stranded RNA
